# The Role of Cardiac Magnetic Resonance Imaging in the Management of Hypertrophic Cardiomyopathy

**DOI:** 10.3390/jcdd12050189

**Published:** 2025-05-15

**Authors:** Luca Pugliese, Alessandra Luciano, Marcello Chiocchi

**Affiliations:** 1 Department of Medical Surgical Sciences and Translational Medicine, University of Rome La Sapienza, Radiology Unit, Sant’Andrea University Hospital, 00189 Rome, Italy; 2 Department of Biomedicine and Prevention, University of Rome Tor Vergata, Unit of Diagnostic Imaging, Policlinico Tor Vergata, 00133 Rome, Italy; alessandraluciano.med@outlook.it (A.L.); marcello.chiocchi@gmail.com (M.C.)

**Keywords:** hypertrophic cardiomyopathy, cardiac magnetic resonance imaging, left ventricular hypertrophy, left ventricular outflow tract obstruction, myocardial fibrosis

## Abstract

Hypertrophic cardiomyopathy (HCM) is the most common genetic cardiomyopathy, caused by either sarcomere protein or other gene mutations. It is a complex and highly heterogeneous disorder, with phenotypes ranging from asymptomatic to severe disease, characterized by asymmetric left ventricular (LV) hypertrophy unexplained by loading conditions, which is also associated with myocardial fiber disarray, and preserved or increased ejection fraction without LV dilation. Comprehensive personal and family history, physical examination, and ECG testing raise suspicion of HCM, and echocardiogram represents the first-line imaging modality for confirming a diagnosis. Moreover, contrast-enhanced cardiac magnetic resonance (CMR) imaging has increasingly emerged as a fundamental diagnostic and prognostic tool in HCM management. This article reviews the role of CMR in HCM identification and differentiation from phenotypic mimics, characterization of HCM phenotypes, monitoring of disease progression, evaluation of pre- and post-septal reduction treatments, and selection of candidates for implantable cardioverter-defibrillator. By providing information on cardiac morphology and function and tissue characterization, CMR is particularly helpful in the quantification of myocardial wall thickness, the detection of hypertrophy in areas blind to echocardiogram, subtle morphologic features in the absence of LV hypertrophy, myocardial fibrosis, and apical aneurysm, the evaluation of LV outflow tract obstruction, and the assessment of LV function in end-stage dilated HCM.

## 1. Introduction

Hypertrophic cardiomyopathy (HCM) is a genetic cardiomyopathy characterized by left ventricular (LV) hypertrophy. It has long been considered a rare and mostly serious disease with limited treatment options, predominantly affecting young males from the Western world [1]. However, in recent decades, the introduction of echocardiography has revealed that HCM is indeed a relatively common disorder with worldwide distribution and a wide age range, that affects both sexes and presents with a broad disease spectrum, from asymptomatic to severe [1]. Moreover, diagnostic and therapeutic innovations have become available, thus substantially decreasing morbidity and mortality from HCM and making it a treatable condition compatible with normal longevity [2]. This article will review the role of cardiac magnetic resonance (CMR) as a fundamental diagnostic and prognostic tool in HCM management, complementary to echocardiography.

## 2. Multifaceted Nature of Hypertrophic Cardiomyopathy (HCM)

The increasing recognition of the existence of multiple HCM phenotypes, from cases with subtle disease without significant disability to symptomatic forms eventually causing arrhythmic sudden cardiac death (SCD), atrial fibrillation (AF) and stroke, and heart failure, has made HCM the most common genetic cardiomyopathy with an estimated prevalence in the general population of 1:200 to 1:500 [3].

Though HCM is often inherited as a Mendelian autosomal dominant disease with variable penetrance and expression, variants in genes encoding sarcomere proteins involved in contractile function are found only in 30–40% of cases [4]. Several mutations of sarcomere genes have been identified so far. The two most common genes are those encoding for beta myosin heavy chain 7 (*MYH7*) and myosin-binding protein C3 (*MYBPC3*), while genes encoding for *TNNI3*, *TNNT2*, *TPM1*, *MYL2*, *MYL3*, and *ACTC1* each account for ≤5% of patients. Although these eight variants show the strongest evidence for association with HCM [5], they do not always predict the clinical phenotype and prognosis, thus suggesting that the heterogeneity of HCM cannot be explained solely by sarcomere gene mutations [6]. The introduction of high-throughput sequencing techniques has also allowed the identification of non-sarcomere gene variants showing a moderate-to-strong association with HCM [4], and cases with autosomal recessive inheritance of these gene variants have been described, especially in populations with a higher degree of consanguinity [7].

The typical pathological feature of HCM is LV hypertrophy, which is predominantly asymmetric and involves the basal interventricular septum below the aortic valve, together with the LV free wall, and less frequently other LV segments and even the right ventricle (RV) [8]. Other features include elongation of the mitral valve leaflet(s) and abnormal insertion of the corresponding papillary muscle [9]. At the histological level, myocyte enlargement and disarray are often associated with various degrees of interstitial fibrosis [10].

Hypertrophic LV is characterized by increased wall thickness at the end-diastole (≥15 mm) at any site, but mostly at the basal anterior septal region (classical HCM pattern), with a ratio of septal to inferolateral wall thickness ≥ 1.3, associated with normal or small cavity and normal end-diastolic and reduced end-systolic volume. Less common variants include the reverse septal, neutral (uniform hypertrophic septum), midventricular, apical, and concentric hypertrophy [11,12]. The LV is typically hyperdynamic with high-normal or elevated ejection fraction [8]. Diastolic dysfunction occurs frequently in HCM as a consequence of interstitial fibrosis and the increased stiffness of the thickened LV wall [1,8]. Approximately 60–70% of individuals with HCM have LV outflow tract obstruction (LVOTO), with half of them exhibiting it at rest and the other half when provoked by stress [13,14]. It is dynamic in nature since it is favored by systolic anterior motion (SAM) and other abnormalities of the mitral valve that abut and obstruct the outflow tract at the subaortic level during systole [8]. Relatively mild segmental hypertrophy (13–15 mm) has been observed in some individuals, associated with normal LV mass and often with dynamic LVOTO [15]. The presence of LVOTO [16], as well as of concomitant severe coronary atherosclerosis [17], may exacerbate the mismatch between myocardial oxygen supply and demand, thus causing myocardial ischemia [18]. Decreased oxygen supply is due to microvascular dysfunction/injury resulting from structural (medial hypertrophy, intimal hyperplasia, and reduced capillary density) and functional abnormalities of the small vessels impairing coronary flow reserve, whereas increased oxygen demand is due to myocardial hypertrophy, with compression from diastolic dysfunction further reducing oxygen delivery [19].

Many HCM patients are asymptomatic or only mildly symptomatic and have a benign course with an extended lifespan. In these individuals, current guidelines for HCM management have even removed the universal restriction from vigorous physical activity or competitive sports, which can be considered following an annual comprehensive evaluation and discussion of the potential benefits and risks with an HCM expert [20].

**Figure 1:**
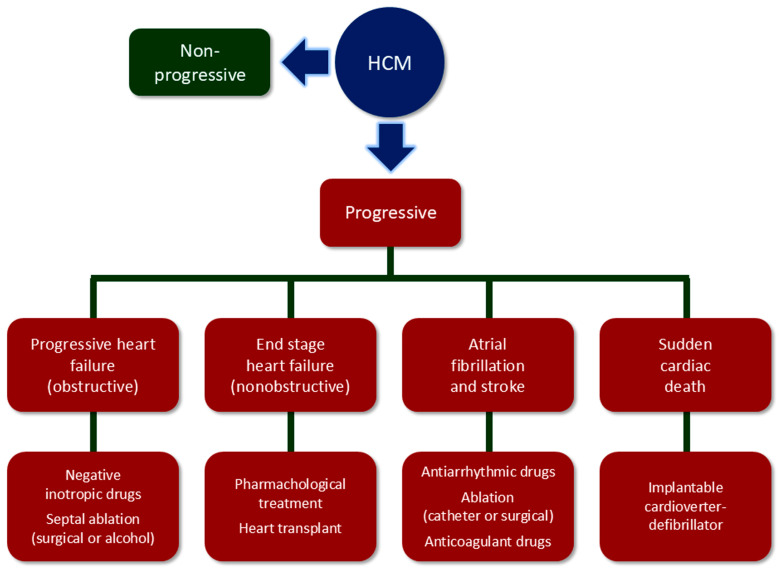
Clinical pictures of HCM and treatment of adverse events. HCM = hypertrophic cardiomyopathy.

However, other individuals experience unfavorable disease progression along one or more of four adverse pathways, although progression along two or three pathways occurs in only 10% of patients [21]. The clinical manifestations are the consequence of arrhythmias or systolic dysfunction and include SCD due to ventricular tachycardia or fibrillation, thromboembolic stroke due to AF, progressive heart failure in individuals with obstructive HCM, and advanced heart failure and end-stage in individuals with non-obstructive HCM [2,3,22]. It has been estimated that these four pathways account for 6%, 39%, 17%, and 4% of HCM cases, respectively, whereas the remaining present a benign or stable course [21,23] (Figure 1).

The prognosis of symptomatic HCM patients has significantly improved over the last decades due to the availability of effective preventive and therapeutic measures. Prophylactic use of intravenous implantable cardioverter-defibrillator (ICD), guided by risk stratification algorithms for candidate selection, is highly effective for preventing SCD and has been the main contributor to the observed ≥10-fold decrease in HMC-related mortality to 0.5% per year [2,3,24]. Invasive septal reduction treatments, including surgical septal myectomy and percutaneous alcohol septal ablation, are successful in relieving symptoms and blocking heart failure progression due to severe LVOTO, which is resistant to negative inotropic drugs (i.e., β-blockers, verapamil, disopyramide, and eventually cardiac myosin inhibitors) [25,26,27]. Atrial fibrillation may be anticipated by using a predictive model [28] and recurrent episodes may be reduced by ~50% with antiarrhythmic drugs and catheter or surgical ablation [29]. Moreover, prophylactic anticoagulant treatment is effective in reducing the risk of thromboembolic events [30]. Finally, even end-stage in patients with non-obstructive HCM, which is now responsible for most deaths, can be controlled using drugs, implantable defibrillators, cardiac resynchronization, and heart transplant [31].

Early detection and risk stratification, together with regular follow-up, are therefore essential for the timely adoption of appropriate measures for the successful prevention and treatment of HCM-related morbidity and mortality.

## 3. Diagnosis, Risk Stratification, and Follow-Up of HCM

**Figure 2:**
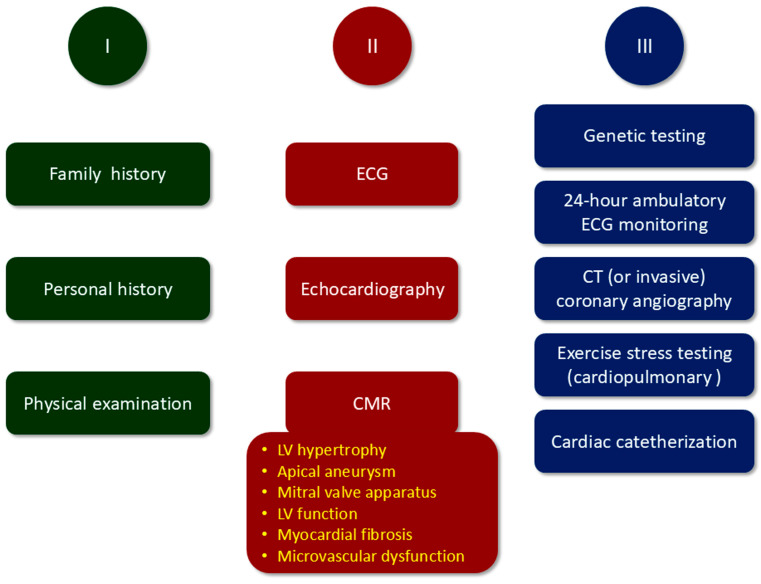
Multi-step diagnostic workup in patients with suspected HCM. HCM = hypertrophic cardiomyopathy; ECG = electrocardiogram; CMR = cardiac magnetic resonance; CT = computed tomography; LV = left ventricular.

Suspicion of HCM may arise from the presence of a family history of HCM or SDC, typical signs and symptoms, or abnormal results of a routine electrocardiogram (ECG) or echocardiography performed for other indications. According to current guidelines for HCM management [20], initial evaluation should indeed include a comprehensive personal and family history and physical examination, followed by a 12-lead ECG and cardiac imaging (Figure 2).

A detailed family history should include at least three generations. In addition, genetic testing is important for facilitating the identification of family members at risk of developing HCM and for diagnosing patients with an atypical clinical presentation [32,33].

When present, symptoms include exertional dyspnea, exercise intolerance, orthopnea, palpitations, presyncope, and syncope, which may be exertional or immediately post-exertional in the case of significant LVOTO, and chest pain [8].

Physical examination should be performed at rest and after maneuvers such as Valsalva and squat-to-stand, in order to detect signs of LVOTO. Signs include a harsh crescendo–decrescendo systolic murmur, prominent apical point of maximal impulse, abnormal carotid pulse, and a fourth heart sound.

The ECG is often abnormal, even in patients with no or only mild LVOTO. Abnormalities comprise voltage changes suggestive of LV hypertrophy and eventually evidence of left atrial enlargement, ST segment and T wave abnormalities, and deep Q waves [8].

Two-dimension (2D) transthoracic echocardiography is the first-line imaging modality for confirming HCM diagnosis and identifying the disease phenotype, thus allowing risk stratification [34,35]. Indeed, it allows us to determine the presence, extent, and pattern of LV hypertrophy, the hallmark of HCM, and also to detect apical aneurysms, assess the presence and severity of LVOTO, and evaluate LV systolic and diastolic and mitral valve function. Provocative maneuvers, such as Valsalva or squat-to-stand (stress echocardiography), should be performed if the resting gradient is <50 mm Hg, to unmask the presence of LVOTO, as it is not significant at rest in half of patients presenting with this complication [36]. Screening with ECG and 2D echocardiography is indicated also in asymptomatic family members of HCM patients [20].

By providing complementary information and as an alternative to echocardiography for patients with inconclusive echocardiogram, CMR imaging has increasingly emerged as a fundamental diagnostic and prognostic tool in HCM management [21].

Other diagnostic procedures are also needed for further characterization of HCM phenotype (Figure 2). If echocardiography is not diagnostic and CMR imaging is unavailable, cardiac computed tomography (CT) may be considered [20], whereas CT (or invasive) coronary angiography is recommended in patients with myocardial ischemia and those at risk for coronary atherosclerosis before surgical septal myectomy [20]. A 24-h ambulatory ECG monitoring is recommended in HCM patients with arrhythmias and at risk for SCD, whereas exercise stress testing (or cardiopulmonary exercise stress testing) is indicated to determine functional capacity [20]. Finally, if the presence or severity of LVOTO is not adequately assessed or the hemodynamic profile is not sufficiently characterized by noninvasive imaging studies, invasive hemodynamic assessment with cardiac catheterization is recommended [20].

Medical history, physical examination, ECG, 2D transthoracic echocardiography, and CMR should be repeated at variable intervals, depending on the presence and severity of symptoms and signs, to monitor the course of the disease [20].

## 4. Cardiac Magnetic Resonance (CMR) Features of HCM

High-field scanners, such as 1.5 T or 3 T, are required for performing CMR. End-expiration breath-hold images are acquired with ECG triggering, and heart long and short axes are localized with scout sequences. Short-axis slices of the LV from the mitral valve to the apex and two-, three- and four-chamber long-axis views of the heart are then acquired and sequences are repeated after the administration of a gadolinium-based contrast agent [37]. The CMR acquisition protocol and sequences, according to the Society for Cardiovascular Magnetic Resonance guidelines [38], are reported in Table 1. Application of a deep learning algorithm may improve the performance of CMR for HCM diagnosis [39], especially if combined with radiomics [40].

By the use of several techniques, CMR has the advantages over echocardiography of a high spatial and temporal resolution, the provision of tomographic images of the heart without a limited view, and the unique ability to identify myocardial fibrosis [41]. Cine-balanced steady-state free precession (SSFP) imaging allows an accurate evaluation of cardiac morphology and function [42]. Late gadolinium enhancement (LGE) CMR imaging using a segmented inversion recovery pulse sequence is able to differentiate between normal and infarcted or fibrotic myocardium [43]. T1 and T2 mapping CMR imaging with end-diastolic Modified Look-Locker Inversion Recovery (MOLLI) sequences are also able to detect areas of myocardial injury without injection of gadolinium-based contrast agents through the assessment of the total extent of expanded extracellular space. Native T1 values are increased in areas of myocardial scarring and interstitial fibrosis and T1 mapping before and after contrast medium injection allows calculation of the extracellular volume fraction [43]. T2-weighted imaging with double inversion recovery black blood preparation can identify areas of myocardial edema or inflammation and T2 mapping can confirm myocardial hyperintensity [44]. Abnormal T1 and T2 values may be observed in areas with or without LGE [41]. Less used techniques include cine phase contrast, perfusion, and tagging CMR imaging, which may serve for the assessment of blood flow and evaluation of myocardial wall motion and strain [41].

**Figure 3:**
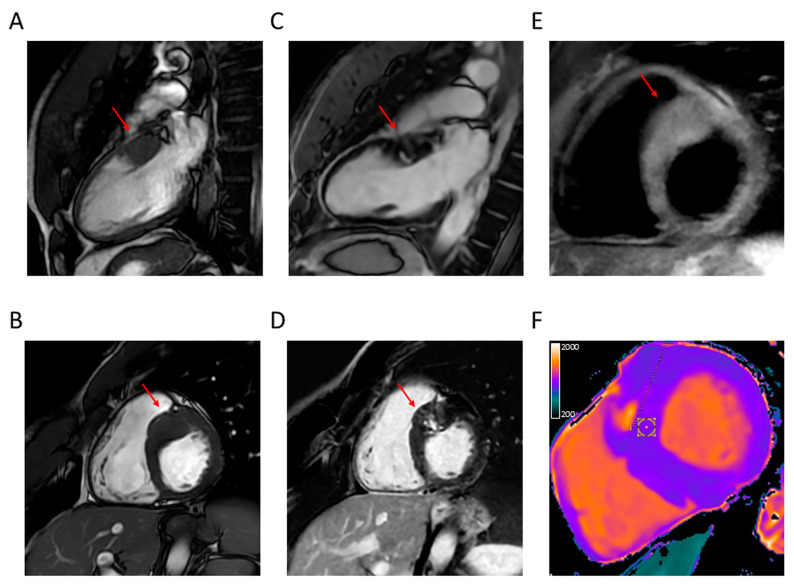
Representative CMR images from a male patient aged 26 years with typical asymmetric basal septal hypertrophy associated with fibrosis. (**A**) two-chamber long axis and (**B**) short axis view showing asymmetric basal septal hypertrophy (arrow); (**C**) two-chamber long axis and (**D**) short axis view showing LGE with patchy distribution at the level of the hypertrophic segment (arrow); (**E**) short axis T2-weighted view showing hyperintensity at the same level (arrow); and (**F**) short axis view showing increased T1 mapping at the same level. CMR = cardiac magnetic resonance; LGE = late gadolinium enhancement.

**Figure 4:**
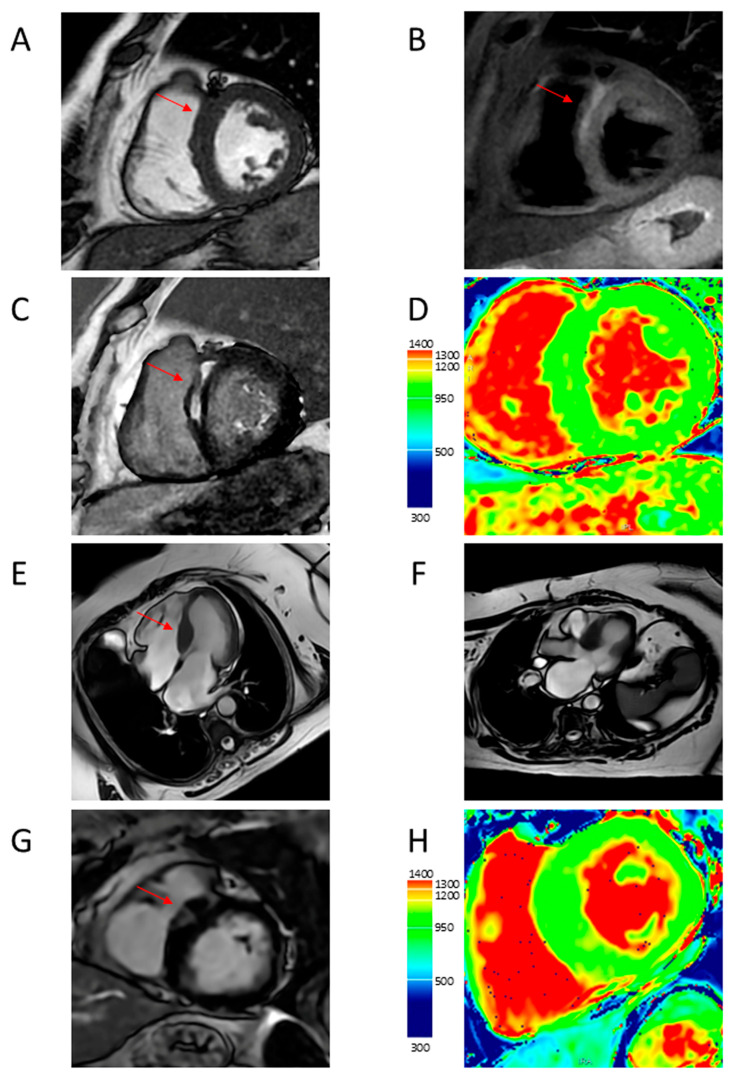
Representative CMR images from a male patient aged 69 years with asymmetric basal septal hypertrophy associated with edema and fibrosis (**A**–**D**) and a male patient aged 80 years with asymmetric basal septal hypertrophy associated with LVOTO and fibrosis. (**A**) Short axis view showing anterior basal septal hypertrophy (arrow); (**B**) short-axis T2-weighed view showing hyperintensity at the same level (arrow); (**C**) short axis view showing LGE with mesocardial distribution at the same level (arrow); (**D**) short axis view showing increased T1 mapping values at the same level; (**E**) four-chamber long axis view showing basal septal hypertrophy (arrow); (**F**) three-chamber long axis view showing LVOTO; (**G**) short axis view showing LGE with patchy distribution at the basal septal level (arrow); and (**H**) short axis view showing increased T1 mapping values at the same level. CMR = cardiac magnetic resonance; LVOTO = left ventricular outflow tract obstruction; LGE = late gadolinium enhancement.

**Figure 5:**
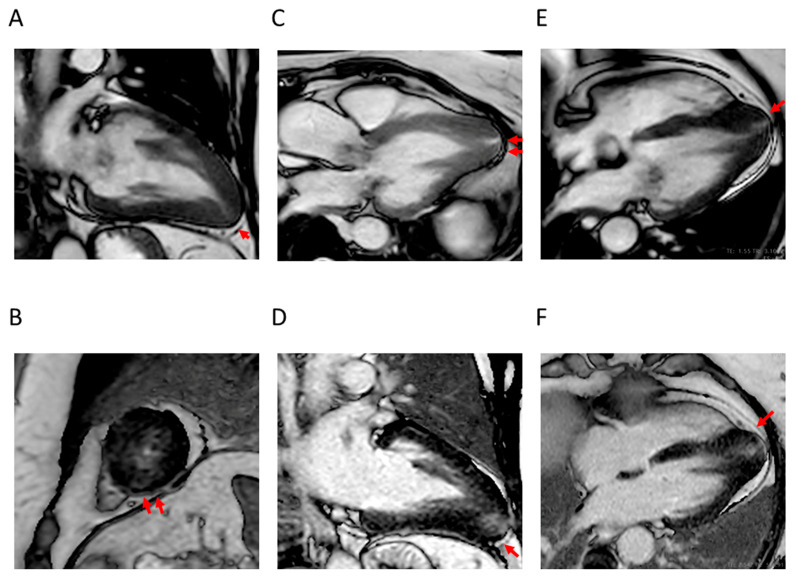
Representative CMR images for a female patient aged 69 years with apical aneurysm. (**A**) two-chamber, (**B**) three-chamber, and (**C**) four-chamber long axis view of the apical aneurysm (arrows); (**D**) short axis view showing LGE with patchy distribution at the level of the apical segment (arrows); (**E**) two-chamber, and (**F**) four-chamber long axis view showing apical LGE (arrow). CMR = cardiac magnetic resonance; LGE = late gadolinium enhancement.

**Figure 6:**
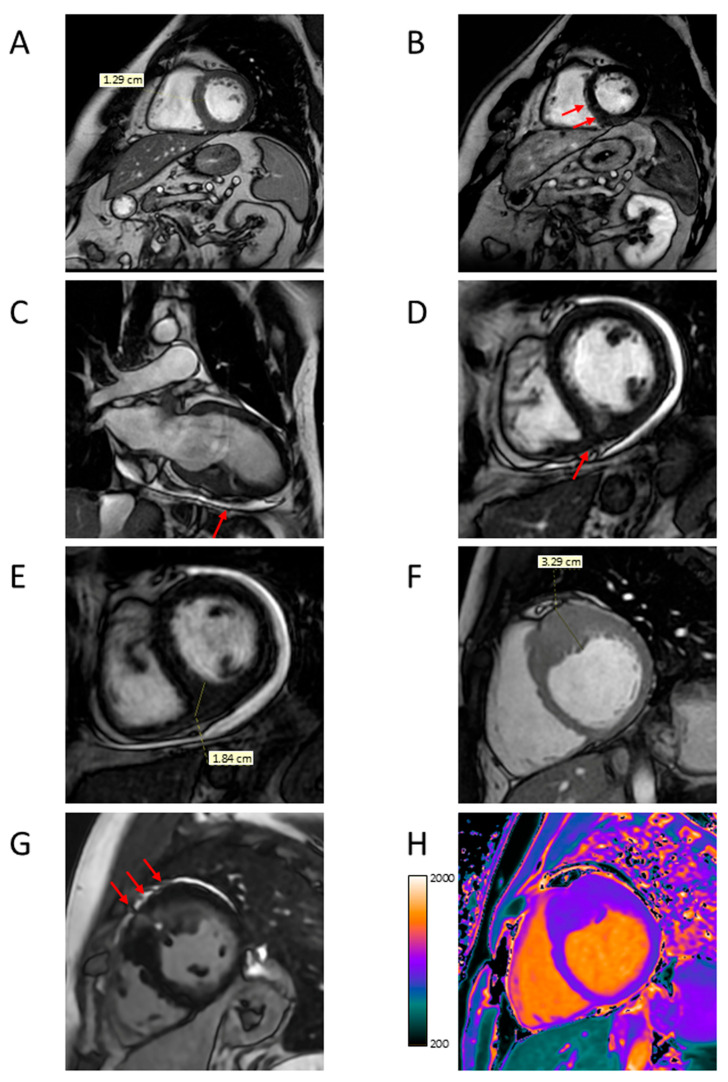
Representative CMR images from a male patient aged 44 years without significant hypertrophy (**A**,**B**), a male patient aged 55 years with moderate asymmetric hypertrophy (**C**–**E**), and a male patient aged 27 years with massive asymmetric hypertrophy (**F**–**H**). (**A**) short axis view showing basal septum with measure of wall thickness; (**B**) short axis view showing LGE with patchy distribution at the basal septal level (arrows); (**C**) two-chamber long axis and (**D**) short axis view showing a crypt at the mid-cavity anterior septal level (arrow); (**E**) short axis view showing mid-cavity anterior septal hypertrophy with measure of wall thickness; (**F**) short axis view showing anterior basal septal hypertrophy with measure of wall thickness; (**G**) short axis view showing LGE at the anterior basal septal level (arrows); and (**H**) short axis view showing increased T1 mapping values at the same level. CMR = cardiac magnetic resonance; LGE = late gadolinium enhancement.

**Figure 7:**
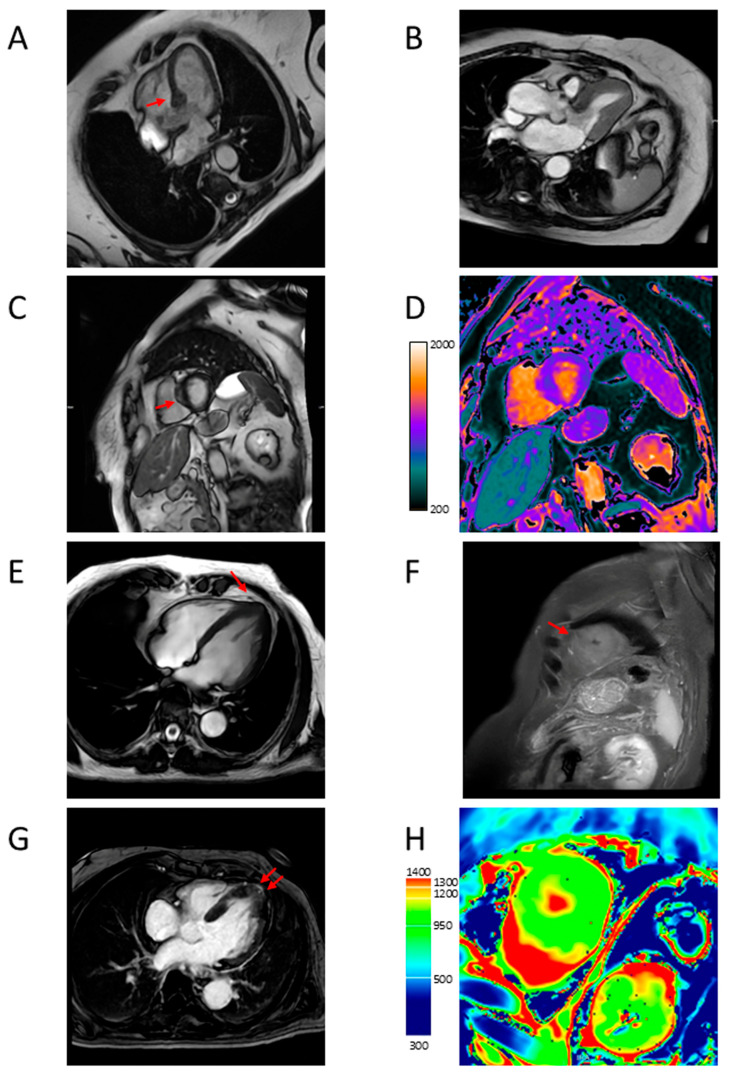
Representative CMR images from a female patient aged 74 years with mass-like hypertrophy (**A**–**D**) and a male patient aged 80 years with apical hypertrophy (**E**–**H**). (**A**) four-chamber long axis view showing mass-like asymmetric basal septal hypertrophy (arrow); (**B**) three-chamber long axis view showing LVOTO; (**C**) short axis view showing LGE with patchy distribution at the level of the hypertrophic segment (arrow); (**D**) short axis view showing increased T1 mapping values at the same level; (**E**) four-chamber long axis view showing asymmetric apical hypertrophy (arrow); (**F**) short axis T2-weighted view showing apical hyperintensity (arrow); (**G**) four-chamber long axis view showing apical LGE (arrows); (**H**) short axis view showing increased T1 mapping values at the same level. CMR = cardiac magnetic resonance; LVOTO = left ventricular outflow tract obstruction; LGE = late gadolinium enhancement.

These characteristics make CRM superior to echocardiography for the identification of LV hypertrophy, apical aneurysm, and structural abnormalities of the mitral valve and subvalvular apparatus contributing to LVOTO and the evaluation of LV function, in addition to allowing the assessment of myocardial fibrosis and microvascular dysfunction (Figure 3, Figure 4, Figure 5, Figure 6 and Figure 7).

### 4.1. LV Hypertrophy

Due to its ability to provide a sharp contrast between the blood pool and myocardium and to distinguish LV from RV and other structures, cine-balanced SSFP CMR imaging with retrospective gating allows an accurate measurement of LV wall thickness, together with chamber size and mass [45].

In addition, because of its no-limited view, it allows the detection of hypertrophy in areas blind to echocardiogram, such as the apex, midventricular region, posterior septum, anterolateral free wall, and RV, as well as asymmetric hypertrophy with a spiral configuration and mass-like focal hypertrophy [46,47,48,49].

Finally, CMR is capable of identifying subtle morphologic features in the absence of LV hypertrophy, such as narrow blood-filled myocardial crypts, apical pouching or thinning, elongated mitral leaflets, and expanded extracellular space, which are typically seen in genotype positive/phenotype negative individuals [50,51,52,53].

### 4.2. Apical Aneurysm

Apical aneurysm has been related to myocardial ischemia and is associated with either apical hypertrophy, with the typical spade-like configuration of the LV cavity [54], or midventricular hypertrophy [55].

The presence of an LV apical aneurysm often goes unrecognized with echocardiography, although it may be detected with contrast echocardiography [56]. However, CMR is the gold standard for detecting apical aneurysms, which appear as a thin-walled dyskinetic or akinetic segment with a transmural scar and, eventually, the presence of a thrombus in the aneurysm cavity [57], which was shown to be associated with thromboembolic stroke [54,56].

### 4.3. LVOTO

Echocardiography is highly useful in the characterization of dynamic LVOTO by allowing estimation of the peak LV outflow tract gradient and assessment of the role of the mitral valve [20]. However, CMR also provides important insights into LVOTO evaluation [57] by allowing a precise assessment of the LV outflow tract and the abnormalities of the mitral valve apparatus contributing to it [58,59,60].

Cine phase contrast CMR imaging is a valuable technique for the assessment of LVOT severity by allowing the quantification of the blood flow through the LV outflow tract and the estimation of the gradient [61], although this is a cumbersome and time-consuming procedure, and hence, is not often used in the clinical setting [41].

Moreover, CMR is able to detect abnormalities of the mitral valve apparatus that are frequently missed by echocardiography. These abnormalities include elongated mitral valve leaflets and muscle bundles, hypertrophic papillary muscles, an increased number of papillary muscles, and anomalous insertion of the anterior papillary muscle to the mitral valve [52,62,63]. A common alteration in patients with HCM is the anterior and apical displacement of the base of the papillary muscle, usually the antero-lateral one, which causes leaflet slack resulting in SAM of the mitral valve and consequent LVOTO independent of apical hypertrophy [64]. Therefore, it is important to differentiate apical displacement of the papillary muscle from apical hypertrophy, with CRM being the gold standard, although contrast echocardiography can be also useful [65]. However, it has been recently shown that apical displacement of the papillary muscle may precede apical hypertrophy, suggesting a pathogenic link between these two conditions [66].

### 4.4. LV Function

Nowadays, CMR is the gold standard for the assessment of LV function due to its superior reproducibility for volumetric assessment when compared with echocardiography, which is limited by 2D geometric assumptions and the necessity of an adequate acoustic window [67,68].

Assessment of LV function is particularly important for risk stratification and disease monitoring since HCM is usually characterized by normal or supernormal LV ejection fraction, which, however, may decline below 50% in approximately 5–10% of patients who progress to the so-called end-stage (or burn-out) HCM, characterized by LV wall thinning and cavity dilation [69].

### 4.5. Myocardial Fibrosis

The unique ability of CMR to provide an in vivo characterization of myocardial tissue with the detection of interstitial fibrosis by the use of LGE and T1 mapping supports a major diagnostic and prognostic role for this imaging procedure in HCM.

At present, LGE is the gold standard for fibrosis detection [57] and is currently used for risk stratification of HCM [70]. However, while the presence of LGE has been associated with adverse outcomes in HCM [71,72,73], there is no consensus on which of the various methods for LGE quantification should be used. These methods, which include visual assessment, full width at half maximum, or signal intensity cut-off values of 2 to 7 standard deviations exceeding the mean value of the non-injured myocardium [43], have indeed shown to provide different LGE measures [74,75,76], and hence, different prognostic estimates, although a meta-analysis showed comparable accuracy in predicting SCD [77]. Around half of individuals with HCM have LGE, which usually appears as a patchy mid-myocardial area within the segment(s) of maximal LV hypertrophy [78,79]. However, LGE can identify focal, but not diffuse fibrosis, which can be detected only by T1 mapping [43]. This represents a promising CMR technique [80,81] that may prove to be superior to LGE, although its clinical relevance has not yet been established [57]. Moreover, it allows estimation of the extracellular volume (ECV) from post and pre-contrast T1 values of blood and myocardium and the level of hematocrit [82].

### 4.6. Microvascular Dysfunction

The use of CMR perfusion imaging allows the presence and extent of microvascular dysfunction to be identified [19].

Adenosine-stress perfusion defects on CMR were indeed shown in more than 40% of HCM patients [83]. Abnormal perfusion was associated with hypertrophy and LGE, although it was detected even in non-hypertrophic and non-LGE segments [84,85,86].

## 5. Role of CMR Imaging in HCM Management

Due to its ability to assess the main typical features of HCM, CMR offers a valuable contribution to the diagnosis and monitoring of the disease as well as to the differentiation of CMR from phenotypic mimics [87,88]. Moreover, it is useful for the characterization of HCM phenotypes and risk stratification, which is essential for predicting outcomes and making therapeutic decisions, including the use of ICD or septal reduction treatments.

### 5.1. Diagnosis and Differentiation from Phenotypic Mimics

The importance of CMR in HCM diagnosis relies on its ability to identify LV hypertrophy, the hallmark of the disease, in a more reliable and accurate manner than echocardiography. While echocardiography may either underestimate or overestimate LV wall thickness, CMR provides precise measurements and is also able to detect hypertrophy in areas blind to echocardiogram or even pre-hypertrophic lesions [45,46].

For the same reasons; CMR has been increasingly utilized in the screening of first-degree relatives of patients with HCM. Moreover; CMR was found to differentiate a positive from a negative genotype of HCM [89]. Screening should be performed with ECG and echocardiography every 1–3 years during childhood and adolescence and every 3–5 years in adult age; but CMR has also a role due to its ability to identify subtle myocardial abnormalities and LGE in the absence or prior to the presence of LV hypertrophy [20]

The suspicion of HCM is based on the presence of LV hypertrophy disproportionate to loading stimuli, at variance with hypertensive cardiomyopathy and aortic stenosis, but is similar to several conditions that mimic HCM, such as the athlete’s heart and infiltrative cardiomyopathies [90,91,92]. Although distinction from these conditions is based on a comprehensive and multiparametric workup, including the search for extracardiac manifestations, specific laboratory and genetic testing, and eventually myocardial biopsy, CMR provides important clues to differential diagnosis [20], which may also take advantage of the analysis of pressure-strain loop-derived myocardial work by speckle tracking echocardiography [93]. This is because of its ability to accurately detect the presence and pattern of LV hypertrophy and quantify the extent of LV wall thickening [45,46], combined with the identification of myocardial interstitial fibrosis [43]. The asymmetric distribution of LV hypertrophy is the main criterion for distinguishing HCM from most of its phenotypic mimics or phenocopies, but patients with HCM may also have a symmetric pattern [41].

In the athlete’s heart, symmetrical LV hypertrophy can be either eccentric or concentric depending on the type of training (endurance versus strength). This condition can be differentiated from HCM because it typically regresses with deconditioning (>2 mm reduction of LV wall thickness after a detraining period) [94] and is characterized by a diastolic wall thickness to left ventricular end-diastolic volume ratio <0.15 mm/m^2^/mL [95]. Moreover, the presence of areas of myocardial scarring/fibrosis at contrast-enhanced CMR with LGE is not consistent with the athlete’s heart, although a mesocardiac LGE stria has been rarely found at the anterior or posterior interventricular junctions and the insertion point of the trabeculae on the ventricular wall [96]. Finally, T1 mapping and ECV values are decreased instead of increased in the athlete’s heart, due to the predominant increase in the cellular over the extracellular myocardial component.

In hypertensive cardiomyopathy, LV hypertrophy is typically moderate, symmetric, and concentric, may regress with aggressive anti-hypertensive treatment, and is usually not associated with LVOTO due to SAM of the mitral valve, although it may be occasionally present [97,98]. In addition, myocardial crypts are not uncommon [99], whereas LGE is absent [100] or shows a linear or patchy pattern at the septal or inferior wall [41] and native T1 and T2 mapping and ECV are either normal or slightly increased [98,101]. Hypertrophy is symmetric also in aortic stenosis, which can be easily identified by CMR [90].

Infiltrative cardiomyopathies include amyloidosis, sarcoidosis, and glycogen/lysosomal storage diseases, such as Fabry–Anderson disease. In amyloidosis, LV hypertrophy is asymmetric in transthyretin amyloidosis and symmetric in immunoglobulin light-chain amyloidosis. Moreover, a right atrial free wall thickness >6 mm is considered to be a specific feature of cardiac amyloid [102]. Diffuse and subendocardial LGE characterizes the early stages, whereas transmural LGE is observed in the advanced stages of the disease [103]. Native T1 mapping and ECV are typically increased and T2 mapping values are also elevated, reflecting myocardial edema [104]. In sarcoidosis, typical findings differ in the acute (inflammatory) and chronic (fibrotic) phases of the disease. In the acute phase, there is LV hypertrophy with signal hyperintensity on T2-weighted imaging and wall motion abnormalities not corresponding to coronary distribution, whereas in the chronic phase, wall thinning or aneurysm are associated with signal hypointensity on T2-weighted imaging and the presence of LGE [105,106]. The distribution of LGE is typically at the level of the septal and lateral wall of basal segments [106], with a pattern predominantly mid-myocardial or subepicardial (non-ischemic) [107]. In Fabry–Anderson disease, LV hypertrophy is diffuse and asymmetrical and is associated with linear or patchy LGE at the basal-inferolateral mid-wall, decreased native T1 mapping values, at variance with other cardiomyopathies, and increased T2 mapping and ECV values [108,109,110].

### 5.2. Characterization of HCM Phenotypes and Risk Stratification

According to current guidelines [20], patients with HCM, adults, children, or adolescents, should undergo comprehensive, systematic noninvasive SCD risk assessment at initial evaluation and every 1 to 2 years thereafter in order to establish the need for ICD. This evaluation should include a personal history of cardiac arrest or sustained ventricular arrhythmias; personal history of syncope suspected by clinical history to be arrhythmic; family history in a close relative of premature HCM-related SCD, cardiac arrest, or sustained ventricular arrhythmias; maximal LV wall thickness, LV ejection fraction, LV apical aneurysm; non-sustained ventricular arrhythmias on continuous ambulatory ECG monitoring; left atrial diameter; and maximal LV outflow tract gradient.

In those patients with HCM who are not otherwise identified as high risk for SCD or in whom a decision to proceed with ICD placement remains uncertain, CMR imaging should be used for a more precise assessment of maximum LV wall thickness, LV ejection fraction, LV apical aneurysm, and possibly LV outflow tract gradient and for determining the extent of myocardial fibrosis with LGE, which is a strong predictor of ventricular arrhythmias [111]. This recommendation is supported by the demonstration of the prognostic significance of CMR assessment of these parameters [112,113,114,115,116,117,118]. The cutoffs are ≥30 mm maximum LV wall thickness in any segment within the chamber (≥28 mm in individual patients, not established for pediatric patients), <50% for LV ejection fraction, ≥50 mm Hg for LV outflow tract gradient, and ≥15% of LV mass for LGE (not established for pediatric patients), whereas the presence of apical aneurysm is relevant independent of size [20].

This information may be used to calculate the 5-year risk of SCD using an SCD risk algorithm such as that of the American College of Cardiologists American Heart Association, which includes age, family history of SCD, maximum LV wall thickness, left atrial diameter, maximal LV outflow tract gradient, non-sustained ventricular tachycardia, unexplained syncope, apical aneurysm, and extensive LGE [119].

By providing important information on LV outflow tract gradient and mitral valve apparatus as well as on LV systolic function, CMR is useful not only for calculating SCD risk but also for assessing the progression of heart failure due to LVOTO (obstructive) and LV systolic dysfunction (non-obstructive). As mentioned above, CMR plays a critical role in identifying abnormalities of the mitral valve apparatus undetected at echocardiography. The identification of these abnormalities is crucial for choosing septal reduction treatments, since they are corrected by surgical septal myectomy but not by percutaneous alcohol septal ablation [58]. In addition, according to current guidelines [120], CMR represents an important imaging tool in the management of heart failure in patients with HCM. In these individuals, detecting an initial decrease in LV ejection fraction may indeed allow prompt recognition and treatment of this condition.

## 6. Conclusions

Contrast-enhanced CMR has increasingly emerged as an indispensable diagnostic and prognostic tool in patients with suspected or known HCM by providing important insights into the phenotype characterization and risk stratification of these individuals, thus guiding the choice of preventive and therapeutic measures.

In particular, it allows more accurate detection and quantification of cardiac morphological and functional abnormalities than 2D-echocardiography, including LV hypertrophy, apical aneurysm, structural abnormalities of the mitral valve and subvalvular apparatus, and LV dysfunction. In addition, it provides information on the presence and extent of myocardial fibrosis, which represents a powerful predictor of adverse outcomes in HCM due to its major role in the pathogenesis of arrhythmias and systolic dysfunction.

Therefore, in order to better manage HCM and its multifaceted manifestations, the use of CMR in clinical practice should be further expanded, and the ability of CMR measures to predict disease progression and adverse outcomes should be evaluated in more depth.

## Figures and Tables

**Table 1:** CMR acquisition protocol and sequences for HCM according to the Society for Cardiovascular Magnetic Resonance guidelines [38].

**Acquisition Protocol**	
**1. Anatomical features** ***a. Pattern and severity of hypertrophy*** - Regional distribution of hypertrophy (including RV) - Reverse septal curvature - Presence of mid-cavity or LVOT obstruction - Presence and location of clefts or crypts - Maximal wall thickness - Thickness of non-hypertrophied segments ***b. Papillary muscles*** - Hypertrophy - Apical displacement ***c. Cavity size*** - LV (LVEDV, LVEDVI, LVESV, LVESVI, LVM, LVMI) - RV (RVEDV, RVEDVI, RVESV, RVESVI) - LA (diameter and volume) ***d. Presence of other abnormalities*** - Intraventricular thrombus - Apical aneurysm	**2. Functional features** ***a. Ventricular*** - LVEF - RVEF ***b. Mitral valve*** - SAM (turbulence on LVOT, peak systolic LVOT velocity/gradient) - Regurgitation (eccentric jet or not, quantification of regurgitant volume and fraction) ***c. Coronary*** - Vasodilator stress perfusion
	**3. Tissue characterization** ***a. LGE*** - Presence - Extent - Location ***b. Advanced tissue characterization*** - Native T1 and T2 mapping (location and magnitude) - ECV (location and magnitude)
**Sequences**	
1. Cine SSFP	6. Early gadolinium enhancement
2. Tagged and other strain-encoding cine	7. Late gadolinium enhancement
3. T1- and T2-weighted imaging	8. Velocity-encoded/phase contrast
4. Quantitative T1 and T2 mapping	9. MR angiography
5. T2* mapping	10. Myocardial perfusion

CMR = cardiac magnetic resonance; HCM = hypertrophic cardiomyopathy; RV = right ventricle; LV = left ventricle; LVOT = LV outflow tract; LVEDV = LV end-diastolic volume; LVEDVI = LV end-diastolic volume index; LVESV = LV end-systolic volume; LVESVI = LV end-systolic volume index; LVM = LV mass; LVMI = LV mass index; RVEDV = RV end-diastolic volume; RVEDVI = RV end-diastolic volume index; RVESV = RV end-systolic volume; RVESVI = RV end-systolic volume index; LA = left atrium; LVEF = LV ejection fraction; RVEF = RV ejection fraction; SAM = systolic anterior motion; LGE = late gadolinium enhancement; ECV = extracellular volume; SSFP = steady-state free precession.

## Data Availability

Not applicable.

## Abbreviations

The following abbreviations are used in this manuscript:

HCM	hypertrophic cardiomyopathy
LV	left ventricular
CMR	cardiac magnetic resonance
SCD	sudden cardiac death
AF	atrial fibrillation
RV	right ventricle
LVOTO	left ventricular outflow tract obstruction
SAM	systolic anterior motion
ICD	implantable cardioverter-defibrillator
ECG	electrocardiogram
2D	two-dimension
CT	computed tomography
SSFP	steady-state free precession
LGE	late gadolinium enhancement
MOLLI	Modified Look-Locker Inversion Recovery
ECV	extracellular volume

## References

[1] Maron B.J., Rowin E.J., Maron M.S. (2022). Hypertrophic Cardiomyopathy: New Concepts and Therapies. Annu. Rev. Med..

[2] Maron B.J., Rowin E.J., Casey S.A., Maron M.S. (2016). How Hypertrophic Cardiomyopathy Became a Contemporary Treatable Genetic Disease with Low Mortality: Shaped by 50 Years of Clinical Research and Practice. JAMA Cardiol..

[3] Maron B.J. (2018). Clinical Course and Management of Hypertrophic Cardiomyopathy. N. Engl. J. Med..

[4] Lopes L.R., Ho C.Y., Elliott P.M. (2024). Genetics of hypertrophic cardiomyopathy: Established and emerging implications for clinical practice. Eur. Heart J..

[5] Ingles J., Goldstein J., Thaxton C., Caleshu C., Corty E.W., Crowley S.B., Dougherty K., Harrison S.M., McGlaughon J., Milko L.V. (2019). Evaluating the Clinical Validity of Hypertrophic Cardiomyopathy Genes. Circ. Genom. Precis. Med..

[6] Maron B.J., Maron M.S., Maron B.A., Loscalzo J. (2019). Moving Beyond the Sarcomere to Explain Heterogeneity in Hypertrophic Cardiomyopathy: JACC Review Topic of the Week. J. Am. Coll. Cardiol..

[7] Allouba M., Walsh R., Afify A., Hosny M., Halawa S., Galal A., Fathy M., Theotokis P.I., Boraey A., Ellithy A. (2023). Ethnicity, consanguinity, and genetic architecture of hypertrophic cardiomyopathy. Eur. Heart J..

[8] Marian A.J., Braunwald E. (2017). Hypertrophic Cardiomyopathy: Genetics; Pathogenesis; Clinical Manifestations; Diagnosis; and Therapy. Circ. Res..

[9] Maron M.S., Olivotto I., Harrigan C., Appelbaum E., Gibson C.M., Lesser J.R., Haas T.S., Udelson J.E., Manning W.J., Maron B.J. (2011). Mitral valve abnormalities identified by cardiovascular magnetic resonance represent a primary phenotypic expression of hypertrophic cardiomyopathy. Circulation.

[10] Davies M.J., McKenna W.J. (1995). Hypertrophic cardiomyopathy--pathology and pathogenesis. Histopathology.

[11] Goldie F.C., Lee M.M.Y., Coats C.J., Nordin S. (2024). Advances in Multi-Modality Imaging in Hypertrophic Cardiomyopathy. J. Clin. Med..

[12] Neubauer S., Kolm P., Ho C.Y., Kwong R.Y., Desai M.Y., Dolman S.F., Appelbaum E., Desvigne-Nickens P., DiMarco J.P., Friedrich M.G. (2019). Distinct Subgroups in Hypertrophic Cardiomyopathy in the NHLBI HCM Registry. J. Am. Coll. Cardiol..

[13] Braunwald E. (2024). Hypertrophic Cardiomyopathy: A Brief Overview. Am. J. Cardiol..

[14] Maron M.S., Olivotto I., Zenovich A.G., Link M.S., Pandian N.G., Kuvin J.T., Nistri S., Cecchi F., Udelson J.E., Maron B.J. (2006). Hypertrophic cardiomyopathy is predominantly a disease of left ventricular outflow tract obstruction. Circulation.

[15] Rowin E.J., Maron B.J., Chokshi A., Kannappan M., Arkun K., Wang W., Rastegar H., Maron M.S. (2018). Clinical Spectrum and Management Implications of Left Ventricular Outflow Obstruction with Mild Ventricular Septal Thickness in Hypertrophic Cardiomyopathy. Am. J. Cardiol..

[16] Cannon R.O., Rosing D.R., Maron B.J., Leon M.B., Bonow R.O., Watson R.M., Epstein S.E. (1985). Myocardial ischemia in patients with hypertrophic cardiomyopathy: Contribution of inadequate vasodilator reserve and elevated left ventricular filling pressures. Circulation.

[17] Sorajja P., Ommen S.R., Nishimura R.A., Gersh B.J., Berger P.B., Tajik A.J. (2003). Adverse prognosis of patients with hypertrophic cardiomyopathy who have epicardial coronary artery disease. Circulation.

[18] Raphael C.E., Cooper R., Parker K.H., Collinson J., Vassiliou V., Pennell D.J., de Silva R., Hsu L.Y., Greve A.M., Nijjer S. (2016). Mechanisms of Myocardial Ischemia in Hypertrophic Cardiomyopathy: Insights from Wave Intensity Analysis and Magnetic Resonance. J. Am. Coll. Cardiol..

[19] Aguiar Rosa S., Rocha Lopes L., Fiarresga A., Ferreira R.C., Mota Carmo M. (2021). Coronary microvascular dysfunction in hypertrophic cardiomyopathy: Pathophysiology; assessment; and clinical impact. Microcirculation.

[20] Ommen S.R., Ho C.Y., Asif I.M., Balaji S., Burke M.A., Day S.M., Dearani J.A., Epps K.C., Evanovich L., Ferrari V.A. (2024). 2024 AHA/ACC/AMSSM/HRS/PACES/SCMR Guideline for the Management of Hypertrophic Cardiomyopathy: A Report of the American Heart Association/American College of Cardiology Joint Committee on Clinical Practice Guidelines. Circulation.

[21] Rowin E.J., Maron M.S., Chan R.H., Hausvater A., Wang W., Rastegar H., Maron B.J. (2017). Interaction of Adverse Disease Related Pathways in Hypertrophic Cardiomyopathy. Am. J. Cardiol..

[22] Maron B.J., Desai M.Y., Nishimura R.A., Spirito P., Rakowski H., Towbin J.A., Rowin E.J., Maron M.S., Sherrid M.V. (2022). Diagnosis and Evaluation of Hypertrophic Cardiomyopathy: JACC State-of-the-Art Review. J. Am. Coll. Cardiol..

[23] Maron B.J., Maron M.S., Rowin E.J. (2017). Perspectives on the Overall Risks of Living with Hypertrophic Cardiomyopathy. Circulation.

[24] Maron B.J., Rowin E.J., Maron M.S. (2019). Paradigm of Sudden Death Prevention in Hypertrophic Cardiomyopathy. Circ. Res..

[25] Vanderlaan R.D., Woo A., Ralph-Edwards A. (2017). Isolated septal myectomy for hypertrophic obstructive cardiomyopathy: An update on the Toronto General Hospital experience. Ann. Cardiothorac. Surg..

[26] Hodges K., Rivas C.G., Aguilera J., Borden R., Alashi A., Blackstone E.H., Desai M.Y., Smedira N.G. (2019). Surgical management of left ventricular outflow tract obstruction in a specialized hypertrophic obstructive cardiomyopathy center. J. Thorac. Cardiovasc. Surg..

[27] Sorajja P. (2017). Alcohol Septal Ablation for Obstructive Hypertrophic Cardiomyopathy: A Word of Balance. J. Am. Coll. Cardiol..

[28] Carrick R.T., Maron M.S., Adler A., Wessler B., Hoss S., Chan R.H., Sridharan A., Huang D., Cooper C., Drummond J. (2021). Development and Validation of a Clinical Predictive Model for Identifying Hypertrophic Cardiomyopathy Patients at Risk for Atrial Fibrillation: The HCM-AF Score. Circ. Arrhythm. Electrophysiol..

[29] Boll G., Rowin E.J., Maron B.J., Wang W., Rastegar H., Maron M.S. (2020). Efficacy of Combined Cox-Maze IV and Ventricular Septal Myectomy for Treatment of Atrial Fibrillation in Patients with Obstructive Hypertrophic Cardiomyopathy. Am. J. Cardiol..

[30] Maron B.J., Olivotto I., Bellone P., Conte M.R., Cecchi F., Flygenring B.P., Casey S.A., Gohman T.E., Bongioanni S., Spirito P. (2002). Clinical profile of stroke in 900 patients with hypertrophic cardiomyopathy. J. Am. Coll. Cardiol..

[31] Argirò A., Zampieri M., Marchi A., Cappelli F., Del Franco A., Mazzoni C., Cecchi F., Olivotto I. (2023). Stage-specific therapy for hypertrophic cardiomyopathy. Eur. Heart J. Suppl..

[32] Ho C.Y., Day S.M., Ashley E.A., Michels M., Pereira A.C., Jacoby D., Cirino A.L., Fox J.C., Lakdawala N.K., Ware J.S. (2018). Genotype and Lifetime Burden of Disease in Hypertrophic Cardiomyopathy: Insights from the Sarcomeric Human Cardiomyopathy Registry (SHaRe). Circulation.

[33] Ahmad F., McNally E.M., Ackerman M.J., Baty L.C., Day S.M., Kullo I.J., Madueme P.C., Maron M.S., Martinez M.W., Salberg L. (2019). Establishment of Specialized Clinical Cardiovascular Genetics Programs: Recognizing the Need and Meeting Standards: A Scientific Statement from the American Heart Association. Circ. Genom. Precis. Med..

[34] Afonso L.C., Bernal J., Bax J.J., Abraham T.P. (2008). Echocardiography in hypertrophic cardiomyopathy: The role of conventional and emerging technologies. JACC Cardiovasc. Imaging.

[35] Abraham M.R., Abraham T.P. (2024). Role of Imaging in the Diagnosis; Evaluation; and Management of Hypertrophic Cardiomyopathy. Am. J. Cardiol..

[36] Joshi S., Patel U.K., Yao S.S., Castenada V., Isambert A., Winson G., Chaudhry F.A., Sherrid M.V. (2011). Standing and exercise Doppler echocardiography in obstructive hypertrophic cardiomyopathy: The range of gradients with upright activity. J. Am. Soc. Echocardiogr..

[37] Hansen M.W., Merchant N. (2007). MRI of hypertrophic cardiomyopathy: Part I.; MRI appearances. AJR Am. J. Roentgenol..

[38] Hundley W.G., Bluemke D.A., Bogaert J., Flamm S.D., Fontana M., Friedrich M.G., Grosse-Wortmann L., Karamitsos T.D., Kramer C.M., Kwong R.Y. (2022). Society for Cardiovascular Magnetic Resonance (SCMR) guidelines for reporting cardiovascular magnetic resonance examinations. J. Cardiovasc. Magn. Reson..

[39] Fahmy A.S., Rowin E.J., Arafati A., Al-Otaibi T., Maron M.S., Nezafat R. (2022). Radiomics and deep learning for myocardial scar screening in hypertrophic cardiomyopathy. J. Cardiovasc. Magn. Reson..

[40] Zhou H., Li L., Liu Z., Zhao K., Chen X., Lu M., Yin G., Song L., Zhao S., Zheng H. (2021). Deep learning algorithm to improve hypertrophic cardiomyopathy mutation prediction using cardiac cine images. Eur. Radiol..

[41] Amano Y., Kitamura M., Takano H., Yanagisawa F., Tachi M., Suzuki Y., Kumita S., Takayama M. (2018). Cardiac MR Imaging of Hypertrophic Cardiomyopathy: Techniques; Findings; and Clinical Relevance. Magn. Reson. Med. Sci..

[42] Carr J.C., Simonetti O., Bundy J., Li D., Pereles S., Finn J.P. (2001). Cine MR angiography of the heart with segmented true fast imaging with steady-state precession. Radiology.

[43] Ambale-Venkatesh B., Lima J.A. (2015). Cardiac MRI: A central prognostic tool in myocardial fibrosis. Nat. Rev. Cardiol..

[44] Simonetti O.P., Finn J.P., White R.D., Laub G., Henry D.A. (1996). “Black blood” T2-weighted inversion-recovery MR imaging of the heart. Radiology.

[45] Rickers C., Wilke N.M., Jerosch-Herold M., Casey S.A., Panse P., Panse N., Weil J., Zenovich A.G., Maron B.J. (2005). Utility of cardiac magnetic resonance imaging in the diagnosis of hypertrophic cardiomyopathy. Circulation.

[46] Maron M.S. (2012). Clinical utility of cardiovascular magnetic resonance in hypertrophic cardiomyopathy. J. Cardiovasc. Magn. Reson..

[47] Moon J.C., Fisher N.G., McKenna W.J., Pennell D.J. (2004). Detection of apical hypertrophic cardiomyopathy by cardiovascular magnetic resonance in patients with non-diagnostic echocardiography. Heart.

[48] Suzuki J., Shimamoto R., Nishikawa J., Yamazaki T., Tsuji T., Nakamura F., Shin W.S., Nakajima T., Toyo-Oka T., Ohotomo K. (1999). Morphological onset and early diagnosis in apical hypertrophic cardiomyopathy: A long term analysis with nuclear magnetic resonance imaging. J. Am. Coll. Cardiol..

[49] Shuroog J., Canakis J., Khan F.J., Suryanarayana P., Soherwardi S. (2021). A Rare Case of Mass-Like Hypertrophic Cardiomyopathy. Cureus.

[50] Deva D.P., Williams L.K., Care M., Siminovitch K.A., Moshonov H., Wintersperger B.J., Rakowski H., Crean A.M. (2013). Deep basal inferoseptal crypts occur more commonly in patients with hypertrophic cardiomyopathy due to disease-causing myofilament mutations. Radiology.

[51] Kebed K.Y., Al Adham R.I., Bishu K., Askew J.W., Klarich K.W., Oh J.K., Julsrud P.R., Foley T.A., Glockner J.F., Nishimura R.A. (2014). Evaluation of apical pouches in hypertrophic cardiomyopathy using cardiac MRI. Int. J. Cardiovasc. Imaging.

[52] Tarkiainen M., Sipola P., Jalanko M., Heliö T., Laine M., Järvinen V., Häyrinen K., Lauerma K., Kuusisto J. (2016). Cardiovascular magnetic resonance of mitral valve length in hypertrophic cardiomyopathy. J. Cardiovasc. Magn. Reson..

[53] Ho C.Y., Abbasi S.A., Neilan T.G., Shah R.V., Chen Y., Heydari B., Cirino A.L., Lakdawala N.K., Orav E.J., González A. (2013). T1 measurements identify extracellular volume expansion in hypertrophic cardiomyopathy sarcomere mutation carriers with and without left ventricular hypertrophy. Circ. Cardiovasc. Imaging.

[54] Yang K., Song Y.Y., Chen X.Y., Wang J.X., Li L., Yin G., Zheng Y.C., Wei M.D., Lu M.J., Zhao S.H. (2020). Apical hypertrophic cardiomyopathy with left ventricular apical aneurysm: Prevalence, cardiac magnetic resonance characteristics, and prognosis. Eur. Heart J. Cardiovasc. Imaging.

[55] Minami Y., Kajimoto K., Terajima Y., Yashiro B., Okayama D., Haruki S., Nakajima T., Kawashiro N., Kawana M., Hagiwara N. (2011). Clinical implications of midventricular obstruction in patients with hypertrophic cardiomyopathy. J. Am. Coll. Cardiol..

[56] Maron M.S., Finley J.J., Bos J.M., Hauser T.H., Manning W.J., Haas T.S., Lesser J.R., Udelson J.E., Ackerman M.J., Maron B.J. (2008). Prevalence; clinical significance; and natural history of left ventricular apical aneurysms in hypertrophic cardiomyopathy. Circulation.

[57] Rowin E.J., Maron M.S. (2016). The Role of Cardiac MRI in the Diagnosis and Risk Stratification of Hypertrophic Cardiomyopathy. Arrhythm. Electrophysiol. Rev..

[58] Patel P., Dhillon A., Popovic Z.B., Smedira N.G., Rizzo J., Thamilarasan M., Agler D., Lytle B.W., Leverm H.M., Desai M.Y. (2015). Left Ventricular Outflow Tract Obstruction in Hypertrophic Cardiomyopathy Patients Without Severe Septal Hypertrophy: Implications of Mitral Valve and Papillary Muscle Abnormalities Assessed Using Cardiac Magnetic Resonance and Echocardiography. Circ. Cardiovasc. Imaging.

[59] Martin R., Lairez O., Boudou N., Méjean S., Lhermusier T., Dumonteil N., Berry M., Cognet T., Massabuau P., Elbaz M. (2013). Relation between left ventricular outflow tract obstruction and left ventricular shape in patients with hypertrophic cardiomyopathy: A cardiac magnetic resonance imaging study. Arch. Cardiovasc. Dis..

[60] Martínez-Vives P., Cecconi A., Vera A., López-Melgar B., Sanz-García A., Viliani D., Nogales-Romo M.T., Muñiz S.H., Olivera M.J., Caballero P. (2025). Tissue tracking analysis and left ventricular outflow tract obstruction in patients with hypertrophic cardiomyopathy. Magn. Reson. Imaging.

[61] Proctor R.D., Shambrook J.S., McParland P., Peebles C.R., Brown I.W., Harden S.P. (2011). Imaging hypertrophic heart diseases with cardiovascular MR. Clin. Radiol..

[62] Rowin E.J., Maron B.J., Lesser J.R., Rastegar H., Maron M.S. (2013). Papillary muscle insertion directly into the anterior mitral leaflet in hypertrophic cardiomyopathy; its identification and cause of outflow obstruction by cardiac magnetic resonance imaging; and its surgical management. Am. J. Cardiol..

[63] Gommans D.H., Bakker J., Cramer G.E., Verheugt F.W., Brouwer M.A., Kofflard M.J. (2016). Impact of the papillary muscles on cardiac magnetic resonance image analysis of important left ventricular parameters in hypertrophic cardiomyopathy. Neth Heart J..

[64] Rajiah P., Fulton N.L., Bolen M. (2019). Magnetic resonance imaging of the papillary muscles of the left ventricle: Normal anatomy; variants; and abnormalities. Insights Imaging.

[65] Ünlü S., Özden Tok Ö., Avcı Demir F., Papadopoulos K., Monaghan M.J. (2021). Differential diagnosis of apical hypertrophic cardiomyopathy and apical displacement of the papillary muscles: A multimodality imaging point of view. Echocardiography.

[66] Filomena D., Vandenberk B., Dresselaers T., Willems R., Van Cleemput J., Olivotto I., Robyns T., Bogaert J. (2023). Apical papillary muscle displacement is a prevalent feature and a phenotypic precursor of apical hypertrophic cardiomyopathy. Eur. Heart J. Cardiovasc. Imaging.

[67] Grothues F., Smith G.C., Moon J.C., Bellenger N.G., Collins P., Klein H.U., Pennell D.J. (2002). Comparison of interstudy reproducibility of cardiovascular magnetic resonance with two-dimensional echocardiography in normal subjects and in patients with heart failure or left ventricular hypertrophy. Am. J. Cardiol..

[68] Hudsmith L.E., Petersen S.E., Francis J.M., Robson M.D., Neubauer S. (2005). Normal human left and right ventricular and left atrial dimensions using steady state free precession magnetic resonance imaging. J. Cardiovasc. Magn. Reson..

[69] Harris K.M., Spirito P., Maron M.S., Zenovich A.G., Formisano F., Lesser J.R., Mackey-Bojack S., Manning W.J., Udelson J.E., Maron B.J. (2006). Prevalence; clinical profile; and significance of left ventricular remodeling in the end-stage phase of hypertrophic cardiomyopathy. Circulation.

[70] Choudhury L., Mahrholdt H., Wagner A., Choi K.M., Elliott M.D., Klocke F.J., Bonow R.O., Judd R.M., Kim R.J. (2002). Myocardial scarring in asymptomatic or mildly symptomatic patients with hypertrophic cardiomyopathy. J. Am. Coll. Cardiol..

[71] Chan R.H., Maron B.J., Olivotto I., Pencina M.J., Assenza G.E., Haas T., Lesser J.R., Gruner C., Crean A.M., Rakowski H. (2014). Prognostic value of quantitative contrast-enhanced cardiovascular magnetic resonance for the evaluation of sudden death risk in patients with hypertrophic cardiomyopathy. Circulation.

[72] Park Y.J., Park S.J., Kim E.K., Park K.M., Lee S.C., On Y.K., Kim J.S. (2020). Semi-quantitative versus quantitative assessments of late gadolinium enhancement extent for predicting spontaneous ventricular tachyarrhythmia events in patients with hypertrophic cardiomyopathy. Sci. Rep..

[73] Maron M.S., Appelbaum E., Harrigan C.J., Buros J., Gibson C.M., Hanna C., Lesser J.R., Udelson J.E., Manning W.J., Maron B.J. (2008). Clinical profile and significance of delayed enhancement in hypertrophic cardiomyopathy. Circ. Heart Fail..

[74] Spiewak M., Malek L.A., Misko J., Chojnowska L., Milosz B., Klopotowski M., Petryka J., Dabrowski M., Kepka C., Ruzyllo W. (2010). Comparison of different quantification methods of late gadolinium enhancement in patients with hypertrophic cardiomyopathy. Eur. J. Radiol..

[75] Aquaro G.D., Positano V., Pingitore A., Strata E., Di Bella G., Formisano F., Spirito P., Lombardi M. (2010). Quantitative analysis of late gadolinium enhancement in hypertrophic cardiomyopathy. J. Cardiovasc. Magn. Reson..

[76] Mikami Y., Kolman L., Joncas S.X., Stirrat J., Scholl D., Rajchl M., Lydell C.P., Weeks S.G., Howarth A.G., White J.A. (2014). Accuracy and reproducibility of semi-automated late gadolinium enhancement quantification techniques in patients with hypertrophic cardiomyopathy. J. Cardiovasc. Magn. Reson..

[77] Kiaos A., Daskalopoulos G.N., Kamperidis V., Ziakas A., Efthimiadis G., Karamitsos T.D. (2024). Quantitative Late Gadolinium Enhancement Cardiac Magnetic Resonance and Sudden Death in Hypertrophic Cardiomyopathy: A Meta-Analysis. JACC Cardiovasc. Imaging.

[78] Mewton N., Liu C.Y., Croisille P., Bluemke D., Lima J.A. (2011). Assessment of myocardial fibrosis with cardiovascular magnetic resonance. J. Am. Coll. Cardiol..

[79] Mentias A., Raeisi-Giglou P., Smedira N.G., Feng K., Sato K., Wazni O., Kanj M., Flamm S.D., Thamilarasan M., Popovic Z.B. (2018). Late Gadolinium Enhancement in Patients with Hypertrophic Cardiomyopathy and Preserved Systolic Function. J. Am. Coll. Cardiol..

[80] Taylor A.J., Salerno M., Dharmakumar R., Jerosch-Herold M. (2016). T1 Mapping: Basic Techniques and Clinical Applications. JACC Cardiovasc. Imaging.

[81] Puntmann V.O., Peker E., Chandrashekhar Y., Nagel E. (2016). T1 Mapping in Characterizing Myocardial Disease: A Comprehensive Review. Circ. Res..

[82] Haaf P., Garg P., Messroghli D.R., Broadbent D.A., Greenwood J.P., Plein S. (2016). Cardiac T1 Mapping and Extracellular Volume (ECV) in clinical practice: A comprehensive review. J. Cardiovasc. Magn. Reson..

[83] Kim E.K., Lee S.C., Chang S.A., Jang S.Y., Kim S.M., Park S.J., Choi J.O., Park S.W., Jeon E.S., Choe Y.H. (2020). Prevalence and clinical significance of cardiovascular magnetic resonance adenosine stress-induced myocardial perfusion defect in hypertrophic cardiomyopathy. J. Cardiovasc. Magn. Reson..

[84] Ismail T.F., Hsu L.Y., Greve A.M., Gonçalves C., Jabbour A., Gulati A., Hewins B., Mistry N., Wage R., Roughton M. (2014). Coronary microvascular ischemia in hypertrophic cardiomyopathy—A pixel-wise quantitative cardiovascular magnetic resonance perfusion study. J. Cardiovasc. Magn. Reson..

[85] Camaioni C., Knott K.D., Augusto J.B., Seraphim A., Rosmini S., Ricci F., Boubertakh R., Xue H., Hughes R., Captur G. (2020). Inline perfusion mapping provides insights into the disease mechanism in hypertrophic cardiomyopathy. Heart.

[86] Yin L., Xu H.Y., Zheng S.S., Zhu Y., Xiao J.X., Zhou W., Yu S.S., Gong L.G. (2017). 3.0 T magnetic resonance myocardial perfusion imaging for semi-quantitative evaluation of coronary microvascular dysfunction in hypertrophic cardiomyopathy. Int. J. Cardiovasc. Imaging.

[87] Tower-Rader A., Kramer C.M., Neubauer S., Nagueh S.F., Desai M.Y. (2020). Multimodality Imaging in Hypertrophic Cardiomyopathy for Risk Stratification. Circ. Cardiovasc. Imaging.

[88] Soler R., Méndez C., Rodríguez E., Barriales R., Ochoa J.P., Monserrat L. (2018). Phenotypes of hypertrophic cardiomyopathy. An illustrative review of MRI findings. Insights Imaging.

[89] Mushtaq S., Chiesa M., Novelli V., Sommariva E., Biondi M.L., Manzoni M., Florio A., Lampus M.L., Avallone C., Zocchi C. (2024). Role of advanced CMR features in identifying a positive genotype of hypertrophic cardiomyopathy. Int. J. Cardiol..

[90] Hansen M.W., Merchant N. (2007). MRI of hypertrophic cardiomyopathy: Part 2; Differential diagnosis; risk stratification; and posttreatment MRI appearances. AJR Am. J. Roentgenol..

[91] Angelini F., Bocchino P.P., Dusi V., Pidello S., De Ferrari G.M., Raineri C. (2025). From thick walls to clear answers: Approaches to diagnosing hypertrophic cardiomyopathy and its mimics. Eur. Heart. J. Suppl..

[92] Licordari R., Trimarchi G., Teresi L., Restelli D., Lofrumento F., Perna A., Campisi M., de Gregorio C., Grimaldi P., Calabrò D. (2023). Cardiac Magnetic Resonance in HCM Phenocopies: From Diagnosis to Risk Stratification and Therapeutic Management. J. Clin. Med..

[93] de Gregorio C., Trimarchi G., Faro D.C., De Gaetano F., Campisi M., Losi V., Zito C., Tamburino C., Di Bella G., Monte I.P. (2023). Myocardial Work Appraisal in Transthyretin Cardiac Amyloidosis and Nonobstructive Hypertrophic Cardiomyopathy. Am. J. Cardiol..

[94] Caselli S., Maron M.S., Urbano-Moral J.A., Pandian N.G., Maron B.J., Pelliccia A. (2014). Differentiating left ventricular hypertrophy in athletes from that in patients with hypertrophic cardiomyopathy. Am. J. Cardiol..

[95] Petersen S.E., Selvanayagam J.B., Francis J.M., Myerson S.G., Wiesmann F., Robson M.D., Ostman-Smith I., Casadei B., Watkins H., Neubauer S. (2005). Differentiation of athlete’s heart from pathological forms of cardiac hypertrophy by means of geometric indices derived from cardiovascular magnetic resonance. J. Cardiovasc. Magn. Reson..

[96] Fogante M., Agliata G., Basile M.C., Compagnucci P., Volpato G., Falanga U., Stronati G., Guerra F., Vignale D., Esposito A. (2021). Cardiac Imaging in Athlete’s Heart: The Role of the Radiologist. Medicina.

[97] Tadic M., Cuspidi C., Plein S., Milivojevic I.G., Wang D.W., Grassi G., Mancia G. (2021). Comprehensive assessment of hypertensive heart disease: Cardiac magnetic resonance in focus. Heart Fail. Rev..

[98] Zdravkovic M., Klasnja S., Popovic M., Djuran P., Mrda D., Ivankovic T., Manojlovic A., Koracevic G., Lovic D., Popadic V. (2022). Cardiac Magnetic Resonance in Hypertensive Heart Disease: Time for a New Chapter. Diagnostics.

[99] Child N., Muhr T., Sammut E., Dabir D., Ucar E.A., Bueser T., Gill J., Carr-White G., Nagel E., Puntmann V.O. (2014). Prevalence of myocardial crypts in a large retrospective cohort study by cardiovascular magnetic resonance. J. Cardiovasc. Magn. Reson..

[100] Rodrigues J.C., Rohan S., Ghosh Dastidar A., Harries I., Lawton C.B., Ratcliffe L.E., Burchell A.E., Hart E.C., Hamilton M.C., Paton J.F. (2017). Hypertensive heart disease versus hypertrophic cardiomyopathy: Multi-parametric cardiovascular magnetic resonance discriminators when end-diastolic wall thickness ≥15 mm. Eur. Radiol..

[101] Arcari L., Hinojar R., Engel J., Freiwald T., Platschek S., Zainal H., Zhou H., Vasquez M., Keller T., Rolf A. (2020). Native T1 and T2 provide distinctive signatures in hypertrophic cardiac conditions—Comparison of uremic; hypertensive and hypertrophic cardiomyopathy. Int. J. Cardiol..

[102] Fattori R., Rocchi G., Celletti F., Bertaccini P., Rapezzi C., Gavelli G. (1998). Contribution of magnetic resonance imaging in the differential diagnosis of cardiac amyloidosis and symmetric hypertrophic cardiomyopathy. Am. Heart J..

[103] Maceira A.M., Joshi J., Prasad S.K., Moon J.C., Perugini E., Harding I., Sheppard M.N., Poole-Wilson P.A., Hawkins P.N., Pennell D.J. (2005). Cardiovascular magnetic resonance in cardiac amyloidosis. Circulation.

[104] Martinez-Naharro A., Baksi A.J., Hawkins P.N., Fontana M. (2020). Diagnostic imaging of cardiac amyloidosis. Nat. Rev. Cardiol..

[105] Tan J.L., Fong H.K., Birati E.Y., Han Y. (2019). Cardiac Sarcoidosis. Am. J. Cardiol..

[106] Markatis E., Afthinos A., Antonakis E., Papanikolaou I.C. (2020). Cardiac sarcoidosis: Diagnosis and management. Rev. Cardiovasc. Med..

[107] Hulten E., Agarwal V., Cahill M., Cole G., Vita T., Parrish S., Bittencourt M.S., Murthy V.L., Kwong R., Di Carli M.F. (2016). Presence of Late Gadolinium Enhancement by Cardiac Magnetic Resonance Among Patients with Suspected Cardiac Sarcoidosis Is Associated with Adverse Cardiovascular Prognosis: A Systematic Review and Meta-Analysis. Circ. Cardiovasc. Imaging.

[108] De Cobelli F., Esposito A., Belloni E., Pieroni M., Perseghin G., Chimenti C., Frustaci A., Del Maschio A. (2009). Delayed-enhanced cardiac MRI for differentiation of Fabry’s disease from symmetric hypertrophic cardiomyopathy. AJR Am. J. Roentgenol..

[109] Perry R., Shah R., Saiedi M., Patil S., Ganesan A., Linhart A., Selvanayagam J.B. (2019). The Role of Cardiac Imaging in the Diagnosis and Management of Anderson-Fabry Disease. JACC Cardiovasc. Imaging.

[110] Pica S., Sado D.M., Maestrini V., Fontana M., White S.K., Treibel T., Captur G., Anderson S., Piechnik S.K., Robson M.D. (2014). Reproducibility of native myocardial T1 mapping in the assessment of Fabry disease and its role in early detection of cardiac involvement by cardiovascular magnetic resonance. J. Cardiovasc. Magn. Reson..

[111] O’Hara R.P., Prakosa A., Binka E., Lacy A., Trayanova N.A. (2022). Arrhythmia in hypertrophic cardiomyopathy: Risk prediction using contrast enhanced MRI.; T1 mapping; and personalized virtual heart technology. J. Electrocardiol..

[112] Maron M.S., Lesser J.R., Maron B.J. (2010). Management implications of massive left ventricular hypertrophy in hypertrophic cardiomyopathy significantly underestimated by echocardiography but identified by cardiovascular magnetic resonance. Am. J. Cardiol..

[113] Rowin E.J., Maron B.J., Carrick R.T., Patel P.P., Koethe B., Wells S., Maron M.S. (2020). Outcomes in Patients with Hypertrophic Cardiomyopathy and Left Ventricular Systolic Dysfunction. J. Am. Coll. Cardiol..

[114] Hanneman K., Crean A.M., Williams L., Moshonov H., James S., Jiménez-Juan L., Gruner C., Sparrow P., Rakowski H., Nguyen E.T. (2014). Cardiac magnetic resonance imaging findings predict major adverse events in apical hypertrophic cardiomyopathy. J. Thorac. Imaging.

[115] Rowin E.J., Maron B.J., Haas T.S., Garberich R.F., Wang W., Link M.S., Maron M.S. (2017). Hypertrophic Cardiomyopathy with Left Ventricular Apical Aneurysm: Implications for Risk Stratification and Management. J. Am. Coll. Cardiol..

[116] Lee D.Z.J., Montazeri M., Bataiosu R., Hoss S., Adler A., Nguyen E.T., Rakowski H., Chan R.H. (2022). Clinical Characteristics and Prognostic Importance of Left Ventricular Apical Aneurysms in Hypertrophic Cardiomyopathy. JACC Cardiovasc. Imaging.

[117] Weng Z., Yao J., Chan R.H., He J., Yang X., Zhou Y., He Y. (2016). Prognostic Value of LGE-CMR in HCM: A Meta-Analysis. JACC Cardiovasc. Imaging.

[118] Kamp N.J., Chery G., Kosinski A.S., Desai M.Y., Wazni O., Schmidler G.S., Patel M., Lopes R.D., Morin D.P., Al-Khatib S.M. (2021). Risk stratification using late gadolinium enhancement on cardiac magnetic resonance imaging in patients with hypertrophic cardiomyopathy: A systematic review and meta-analysis. Prog. Cardiovasc. Dis..

[119] Ommen S.R., Mital S., Burke M.A., Day S.M., Deswal A., Elliott P., Evanovich L., Hung J., Joglar J.A., Kantor P. (2021). 2020 AHA/ACC guideline for the diagnosis and treatment of patients with hypertrophic cardiomyopathy: A report of the American College of Cardiology/American Heart Association Joint Committee on Clinical Practice Guidelines. J. Thorac. Cardiovasc. Surg..

[120] Heidenreich P.A., Bozkurt B., Aguilar D., Allen L.A., Byun J.J., Colvin M.M., Deswal A., Drazner M.H., Dunlay S.M., Evers L.R. (2022). 2022 AHA/ACC/HFSA Guideline for the Management of Heart Failure: A Report of the American College of Cardiology/American Heart Association Joint Committee on Clinical Practice Guidelines. Circulation.

